# Depletion of Neurocan in the Prefrontal Cortex Impairs Temporal Order Recognition, Cognitive Flexibility and Perisomatic GABAergic Innervation

**DOI:** 10.1007/s10571-026-01762-2

**Published:** 2026-06-09

**Authors:** David Baidoe-Ansah, Hector Carceller, Hadi Mirzapourdelavar, Marta Pérez-Rando, Luisa Strackeljan, Borja Garcia-Vazquez, Constanze Seidenbecher, Rahul Kaushik, Juan Nacher, Alexander Dityatev

**Affiliations:** 1 Molecular Neuroplasticity, German Center for Neurodegenerative Diseases (DZNE), 39120 Magdeburg, Germany; 2 Neuroplasticity Unit, Institute for Biotechnology and Biomedicine (BIOTECMED), University of Valencia, 46100 Valencia, Spain; 3 Leibniz Institute for Neurobiology, 39118 Magdeburg, Germany; 4 Center for Behavioral Brain Sciences (CBBS), 39106 Magdeburg, Germany; 5 Spanish National Network for Research in Mental Health CIBERSAM, 28029 Madrid, Spain; 6 Fundación Investigación Hospital Clínico de Valencia, INCLIVA, 46010 Valencia, Spain; 7 Medical Faculty, Otto-von-Guericke University, 39120 Magdeburg, Germany

**Keywords:** Extracellular matrix, Perineuronal net, Perisomatic inhibition, Neurocan, Cognitive flexibility, Temporal order recognition memory, GABAergic synapse, Glutamatergic synapse

## Abstract

The condensed form of the neural extracellular matrix (ECM), known as perineuronal nets (PNNs), is predominantly associated with parvalbumin-expressing (PV+) interneurons in the cortex and hippocampus. PNNs are enriched in lecticans, including neurocan (Ncan). Genetic variation in the human *Ncan* gene has been linked to alterations in hippocampus-dependent memory, structural differences in the prefrontal cortex (PFC), and increased risk for schizophrenia and bipolar disorder. However, the specific contribution of Ncan to PFC-dependent cognitive functions and synaptic organization remains incompletely understood. Here, we investigated the consequences of region-specific reduction of Ncan in the infralimbic cortex of mice, a region considered homologous to the dorsomedial part of the human medial PFC. Using intracortical adeno-associated virus delivery of shRNA targeting Ncan, we examined PNN-associated markers, synaptic organization, and PFC-dependent behaviors. Ncan knockdown was associated with reduced *Wisteria floribunda agglutinin* (WFA) labeling of PNNs surrounding PV+ interneurons, suggesting alterations in PNN composition. Behaviorally, mice with reduced Ncan expression exhibited mild but consistent impairments in medial PFC–dependent temporal order recognition memory and in aspects of reversal spatial learning in a labyrinth task. At the cellular level, immunohistochemical analyses revealed a reduction in markers associated with perisomatic GABAergic innervation of PV+ interneurons, together with an increase in the density of vGLUT1-immunopositive puncta in the surrounding neuropil. These structural changes were not observed in constitutive Ncan knockout mice, suggesting the presence of compensatory mechanisms in this model. Together, our findings highlight a functional role of Ncan in supporting perisomatic GABAergic innervation, temporal order recognition memory and cognitive flexibility, important cognitive resources impaired in neuropsychiatric disorders.

**Supplementary Information:**

The online version contains supplementary material available at 10.1007/s10571-026-01762-2.

## Introduction

The medial prefrontal cortex (mPFC) has been identified as a critical domain for long-term memory storage (Euston et al. 2012; Hylin et al. 2013) and decision-making (Rushworth et al. 2011; Domenech 2015; Funahashi 2017; Domenech et al. 2020). Additionally, the mPFC contributes significantly to the retrieval phase of memory remodeling (Peters et al. 2013; Hauser et al. 2015) and upon cognitive flexibility (CF) (Coutlee and Huettel 2012; Hauser et al. 2015). The latter is a component of executive function, which includes task-shifting properties (Hauser et al. 2015). It is defined as the ability to adapt to a steady dynamic environment and to filter information to focus on relevant features in an impending task (Monsell 2003; Happel and Frischknecht 2016; Buttelmann and Karbach 2017). Therefore, CF is an essential component of reversal learning paradigms (Izquierdo et al. 2017), which is regulated by the integrity of the neural extracellular matrix (ECM) (Morellini et al. 2010; Happel et al. 2014).

The neural ECM comprises diverse structural and functional families of ECM molecules, including hyaluronic acid, chondroitin sulfate proteoglycans (CSPGs), tenascins, and link proteins. They may form the condensed form of neural ECM, known as perineuronal nets (PNNs), which tightly associate with somata, proximal dendrites, and axon initial segments of parvalbumin-expressing (PV+) interneurons in the cortex and hippocampus (Dityatev and Schachner 2003; Bruckner et al. 2006; Fawcett et al. 2019). PNNs are formed in an activity-dependent manner (Dityatev et al. 2007) and are broadly detectable in the hippocampus, mPFC, cerebellum, and other brain areas (Bruckner et al. 2006; Lupori et al. 2023). PNN formation and maturation coincide with the onset and closure of the critical period that can be reopened by PNN disintegration (Pizzorusso et al. 2002; Hou et al. 2017). Functionally, PNNs have been associated with neural processes such as synaptic plasticity, as well as formation, stabilization, updating, and recall of memory (Dityatev and Fellin 2008; Gogolla et al. 2009; Happel and Frischknecht 2016; Thompson and Chen 2017). Interestingly, PNNs are important regulators of synaptic input to PV+ interneurons, particularly their GABAergic innervation (Carceller et al. 2020).

PNNs are incorporating lecticans, namely aggrecan (Acan), versican (Vcan), brevican (Bcan), and neurocan (Ncan), which all bind to hyaluronan as a backbone and are stabilized by hyaluronan and proteoglycan link proteins (HAPLN1-4), and cross-linked by the glycoprotein tenascin-R (TnR) (Dityatev et al. 2010; Morawski et al. 2014). A study by Suttkus and colleagues dissected the importance of individual ECM molecules in the somatosensory cortex using KO mice and identified Acan, HAPLN1, and TnR as essential contributors to PNN formation (Suttkus et al. 2014). Their findings are in line with other studies (Yamada et al. 2017). Interestingly, a KO study in the medial nucleus of the trapezoid body (MNTB) of mice implicated Ncan in PNN development, potentially via modulation of the expression pattern of other PNN molecules such as HAPLN1 and Bcan (Schmidt et al. 2020).

Ncan, a brain-specific CSPG, affects neuronal adhesion and migration by interacting with the neural cell adhesion molecule NCAM (Raum et al. 2015). Expression of Ncan is downregulated postnatally and then upregulated again in adulthood (Pizzorusso et al. 2002). This pattern of expression correlates with the inhibitory phenotype of mature CSPGs toward axonal growth, which is reversed upon CSPG digestion (Pizzorusso et al. 2002). Similarly, Ncan is upregulated during glial scar formation after injury, where it prevents axonal growth (Asher et al. 2000).

Several previous studies have used *Ncan* KO mice (Zhou et al. 2001; Schmidt et al. 2020) and enzymatic ECM digestion (Shi et al. 2019) to dissect the role of Ncan. However, these approaches have some limitations. There can be activation of compensatory mechanisms, particularly during early development, in KO models (Morawski et al. 2014; El-Brolosy and Stainier 2017), while the global acute digestion of ECM with enzymes such as chondroitinase ABC (chABC) or hyaluronidase (Dityatev et al. 2007; Happel et al. 2014; Yang et al. 2015) affects multiple ECM molecules. Hence, in our previous study, we employed a shRNA-based approach and validated it in vitro (Baidoe-Ansah et al. 2025). Interestingly, it revealed that Ncan depletion altered the ECM architecture around the soma and the axon initial segment (AIS) of inhibitory neurons, thereby elevating neuronal activity (Baidoe-Ansah et al. 2025).

The human *Ncan* gene single-nucleotide polymorphism (SNP) rs1064395 is a well-documented risk factor for schizophrenia and bipolar disorder. Importantly, this risk variant in the Ncan gene is associated with reduced hippocampus-dependent memory function, and variation of PFC structure and ECM composition in healthy humans (Assmann et al. 2020), and in bipolar disorder patients (Cichon et al. 2011), as well as with increased cortical folding in the lateral occipital cortex and the PFC in patients with schizophrenia (Schultz et al., 2014). Miró and colleagues show the association between neurocan and hyperactivity, a subdimension of the mania factor, in Ncan KO mice (Miro et al. 2012). Ncan knockdown abolished the antidepressant effect of ketamine, whereas stress vulnerability was effectively counteracted through the overexpression of Ncan, highlighting its putative role in regulating depressive and anxiety-like behaviors as a PNN component (Yu et al., 2022).

Based on these findings, we hypothesize that Ncan may regulate PFC-dependent cognitive functions. To test this hypothesis and uncover potential molecular mechanisms underlying this effect, we employed a shRNA-based approach (Baidoe-Ansah et al. 2025) and examined mPFC-dependent forms of learning and memory in Ncan shRNA-injected mice. We observed a loss of temporal order recognition memory and impairment in reversal spatial learning in these animals. As a potential cellular mechanism, we observed a significant reduction in the perisomatic GABAergic innervation of PV+ cells in Ncan shRNA-injected mice. A preprint containing major results of this work has been published in BioRxiv (doi: 10.1101/2023.04.18.537277).

## Methods

### Animal Housing and Ethics

All animal experiments were conducted in accordance with the ethical animal research standards defined by German and Spanish law with the Directive 2010/63/EU of the European Parliament and of the Council of 22 September 2010 on the protection of animals used for scientific purposes and approved by the Ethical Committee on Animal Health and Care of the State of Saxony-Anhalt, Germany, with license numbers 42502-2-1322 DZNE and 42502-2-1343 DZNE, or by the Committee on Bioethics of the Universitat de València. This study used a total of 30 male mice including five wild-type and six *Ncan* KO 3-month-old male mice derived from the same founder colonies (Zhou et al. 2001), ten 2- to 3-month-old male C57BL6/J mice injected with Control shRNA AAV (hereafter referred to as Control shRNA), and ten 2- to 3-month-old male C57BL6/J mice injected with Ncan shRNA AAV (hereafter referred to as Ncan shRNA). The number of mice used in each experiment is given in the text and figure legends. Mice used for AAV injections were transferred from the animal breeding house to the research facility and housed individually, with food and water available ad libitum for at least 72 h before experiments, under a reversed 12/12 light/dark cycle (light on 9 PM). All behavioral experiments were performed during the dark phase of the cycle, i.e., when mice are active, under constant temperature and humidity. All wild-type and *Ncan* KO mice were housed in groups of 2 to 4 in a standard environment (12 h light/dark cycle) and with *ad libitum* access to food and water. Every effort was made to minimize the number of animals used and their suffering.

## Stereotaxic Injection of Ncan shRNA and Control shRNA into the mPFC

Here, twenty 2- to 3-month-old mice were injected with AAVs (AAV2/DJ viral particles) for expression of shRNA under the U6 promoter, which has been validated in vitro (Baidoe-Ansah et al. 2025) to deplete Ncan mRNA. The viral particles were obtained by transfection of HEK 293 T cells using PEI (1ng/µl) with an equimolar mixture of the shRNA encoding AAV2 U6 GFP, pHelper (Cell Biolabs Inc., San Diego, CA, USA), and RapCap DJ plasmids (Cell Biolabs Inc., San Diego, CA, USA) and purified filtrates using the pre-equilibrated HiTrap Heparin HP affinity columns (GE HealthCare, Chicago, IL, USA). The mice were randomly assigned to two groups (Control and Ncan shRNA), briefly sedated with isoflurane and fixed to a stereotactic frame (SR-6 M, Narishige Scientific Instrument Lab, Japan). Mice were anesthetized with isoflurane, initially adjusted to 4% for induction, then reduced to 1.5–2%, with oxygen set to 0.4 L/min (Baxter 250 ml Ch.-B.: 17L13A31). The body temperature of mice was maintained at 37 °C using a heating pad (ATC1000 from World Precision Instrument, USA). Ophthalmic ointment (BRAND) was applied to protect the eyes during surgery, after which the skin was cleaned with 70% ethanol, and hair was shaved. A volume of 1000 nl was injected using a 10 µl NanoFil syringe (World Precision Instruments, USA) and a calibrated glass microelectrode connected to an Ultra microinfusion pump (UMP3, World Precision Instruments, USA) at a rate of 3 nl/sec. The coordinates for AAV injection were dorso-ventral (DV) from the brain surface, anterior-posterior (AP) from bregma, and medio-lateral (ML) from the midline (in mm): AP, + 1.77; ML, −0.3; DV, −2.2. Ncan shRNA AAV (3.76 × 10^11^ vg/ml) and Control shRNA AAV (5.86 × 10^11^ vg/ml) were injected bilaterally. After all procedures, the animals were placed in a recovery chamber under red light for 15 min.

## Open Field and Recency Task

**Fig. 1:**
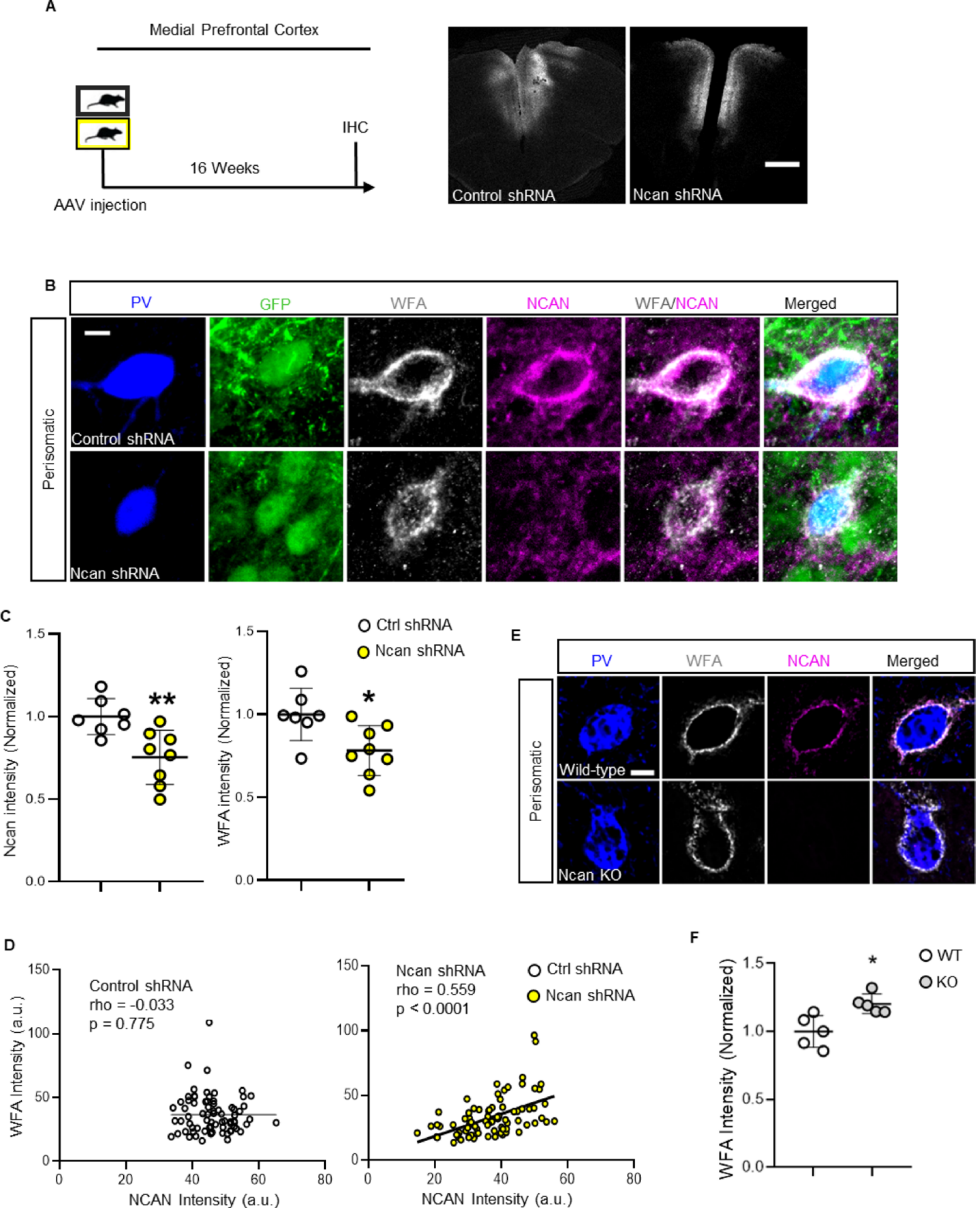
Knockdown of Ncan expression in the PFC affected PNN maintenance. **A** The scheme of experiments and examples of AAV expression 16 weeks after injection in the infralimbic part of the PFC. Scale bar 100 μm. **B** Representative images of PNNs that were visualized and quantified after Ncan knockdown. Scale bar, 5 μm. **C** The expression of WFA and Ncan in perineuronal nets per animal was reduced in Ncan shRNA AAV-injected (*N* = 83 neurons from 9 mice) mice compared to Control shRNA AAV-injected mice (*N* = 74 neurons from 7 mice), Unpaired t-test with Welch’s correction. **D** Correlation between the intensities of Ncan and WFA labelling in individual cells, *rho* is the Spearman coefficient of correlation. **E** Lack of Ncan in PNNs of Ncan KO mice in representative images. Scale bar, 5 μm. **F** Quantification of the mean immunofluorescence intensity of WFA in wild-type (106 neurons from 5 mice) and Ncan KO mice (241 neurons from 6 mice). Unpaired t-test. Bar graphs show mean ± SD values

**Fig. 2:**
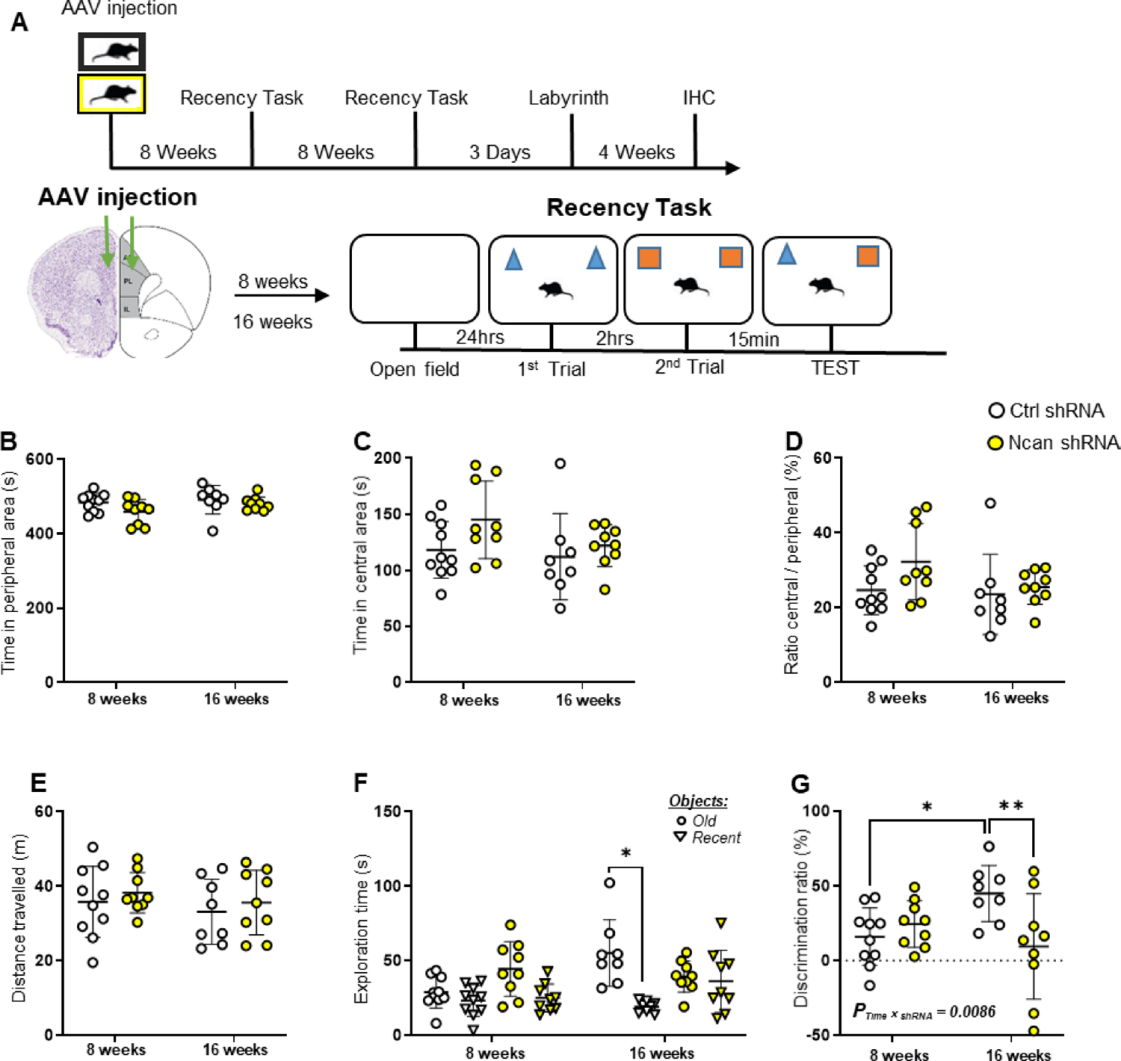
Knockdown of Ncan expression in the PFC impaired temporal order recognition memory. **A** Timeline of behavioral experiments and the recency task performed 8 and 16 weeks after AAV injection. The initial open field test of the recency task showed no changes in time spent in the peripheral **(B)** and central areas **(C)** as well as the ratio between them **(D)**, and the distance travelled **(E)**. The time intervals spent exploring old and recent objects, 8 weeks or 16 weeks after AAV injection, were measured, with a significant effect of Ncan knockdown observed after 16 weeks **(F**,** G)**. Bar graphs show mean ± SD values. *p < 0.05, **p < 0.01, represent significant differences between Control shRNA (*N* = 10) and Ncan shRNA (*N* = 9) 8-week treated mice or Control shRNA (*N* = 8) and Ncan shRNA (*N* = 9) 16-week treated mice using two-way ANOVA with Holm-Sidak’s post-hoc test for all panels except for **(F)** where three-way ANOVA with Holm-Sidak’s post-hoc correction was used

Before behavioral testing, mice were handled and habituated to the behavioral room using the glass tunnel method of handling for 3–4 days (Gouveia and Hurst 2017). On the 5th day, the open-field task was performed, where mice were placed in an empty arena (50 × 30 cm) for 10 min (Baidoe-Ansah et al. 2022; Holter et al. 2015; Kaushik et al. 2018). The positions of animals were tracked using an overhead camera and the tracking software (Anymaze 4.99; Stoelting Co., Wood Dale, IL, USA). The total distance travelled and the time spent in the central vs. peripheral areas were used as indicators of general activity and anxiety. The temporal aspect of learning and memory has been shown to depend on mPFC, a region of the CNS associated with executive function. One behavioral task that can assess this aspect is the temporal order recognition (recency) task (Naya et al. 2017). It was performed 24 h after the open-field task. Before and after each session, the floors and walls were cleaned with 70% ethanol. In each of three sessions, mice were placed at the center of the apparatus using the glass tunnel – initially facing away from two objects – and allowed to explore for 10 min. During the first session, two indistinguishable objects were presented to the mice (First Sampling-phase: objects **A**,** A**). The second set of different objects (Second Sampling-phase: **B**,** B**) was presented after an hour. Fifteen minutes later, a probe test was performed with one object from each training session being presented (**A**, **B**). The time intervals spent exploring these objects (old **A** and recent **B**) were measured by a trained observer who was blinded to treatment conditions (Bonardi et al. 2016). Exploring time for old and recent objects, as well as the discrimination ratio [(old-recent)/(old+recent)] × 100%, was used to evaluate animals’ recognition memory. New pairs of counterbalanced old and recent objects were used for recency tests performed 8 and 16 weeks after AAV injection (Figs. 1 and 2).

## Labyrinth Task (Dry maze)

**Fig. 3:**
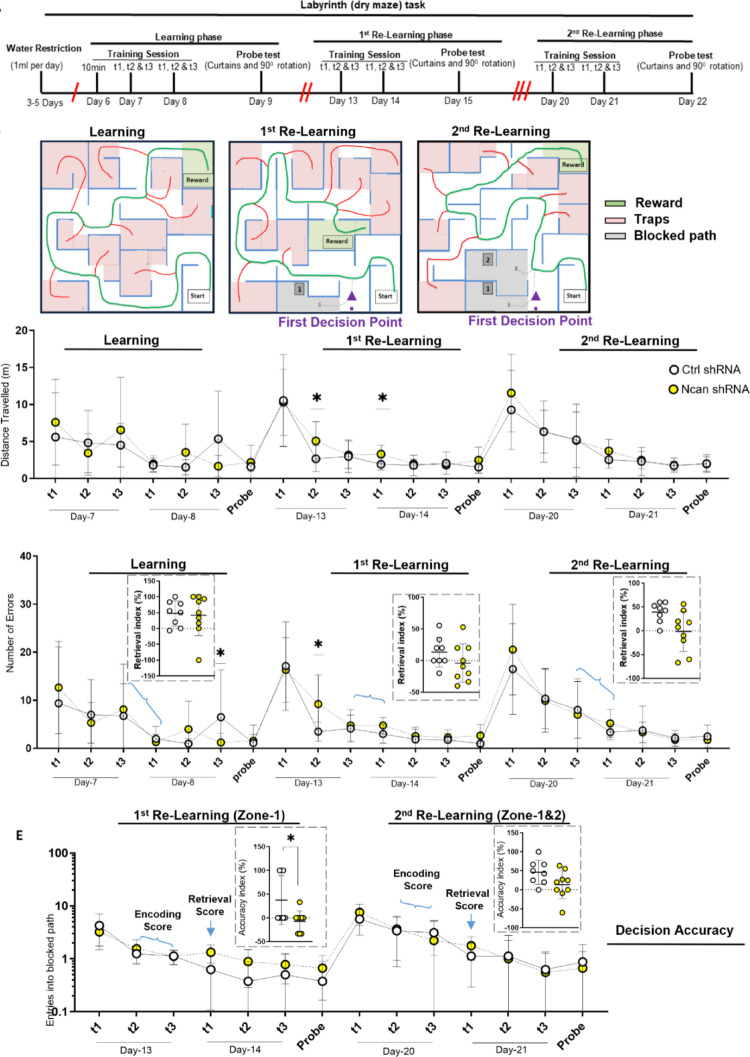
Ncan knockdown impaired consolidation of spatial memory after reversal learning. **A** Timeline for the labyrinth (dry maze) task that was designed to include one phase of learning and two phases of reversal learning. **B** Design of the labyrinths used, pointing to the start zone, a reward location (green zone), traps (pink zones), blocked paths (gray zones) along the right paths (green line), and wrong paths (red lines). The performance of the mice was measured by the distance travelled and the number of errors. **C** No difference in mean distance travelled was observed between Control and Ncan shRNA-treated mice. **D** Ncan shRNA-treated mice potentially failed to consolidate spatial memory during the two relearning phases of the Labyrinth task. **E** Ncan shRNA-treated mice were less accurate at discriminating between the blocked path(s) and the new path to the new reward location. Bar and line graphs show mean ± SD values. *p < 0.05, **p < 0.01, ***p < 0.001, and **** *p* < 0.0001 represent significant differences between Control shRNA (*N* = 8) and Ncan shRNA (*N* = 9) treated mice using two-way RM ANOVA with Fisher’s LSD for all line plots and unpaired t-test with Welch’s correction for all bar plots

The Labyrinth task, also known as the dry maze, is a behavioral setup capable of assessing hippocampus-dependent spatial learning and memory and can also be used to test mPFC-dependent memory retrieval and reversal learning (Peters et al. 2013). This setup is non-stressful compared to the water maze (Harrison and Feldman 2009), yet capable of measuring the various learning strategies mice use for spatial navigation, such as place and response learning, as well as the egocentric strategy (Rich and Shapiro 2007; Peters et al. 2013). Using the labyrinth setup to study mPFC functions, we designed a 3-phase learning paradigm that included an initial learning phase (4 days), 2 relearning phases (3 days each), and a 3-day inter-learning delay (Fig. 3A&B).

Before the labyrinth task, mice were restricted to 1.0 ml of water per day for up to 5 days. On the 6th day (day 1 of the initial-learning-phase), mice were allowed to explore the maze for 10 min with 1.0 ml of water (as a reinforcer) located in the reward zone. Mice were then placed in the maze for 3 trials per day on days 7, 8, 13, 14, 20, and 21, with each trial manually ended upon entry into the reward zone. A 1-hour inter-trial delay was used for each training session. On days 9, 15, and 22, a probe test was performed, which included a 90° rotation and dropped curtains to enclose the maze and exclude extra-maze (distal) cues. This probe test was introduced to assess the use of allocentric vs. egocentric navigation strategies. During the relearning phase, the reward path (Fig. 3B, green line) and the reward location (green zone) were changed while maintaining the start zone. A decision point was generated by blocking the recently learned path to the reward. A learning curve was then measured using an overhead camera with animal-tracking software (Anymaze 4.99; Stoelting Co., Wood Dale, IL, USA). The following parameters were calculated: distance traveled before entry into the reward zone, the number of errors (indicated by the red path line), and latency to reward. For all phases of learning, the start zone was kept constant. During the relearning phases, mice were exposed to a redesigned labyrinth (new path and reward location). This exposure was to measure mPFC function in decision-making, as shown by Peters and colleagues (Peters et al. 2013). The old path was blocked (gray zones; **Zone-1** for 1 st relearning and **Zone-1&2** for 2nd relearning), and the contribution of mPFC to decision-making was assessed at the decision point, i.e., the point of intersection between the old and new paths (Fig. 3A&B).

Moreover, several studies have used within-day and between-day error indices to show neural dissociations for spatial navigation (Churchwell et al. 2010). Therefore, in this study, we used the mean number of errors to measure the between-day retrieval index, which would reflect overnight consolidation of acquired memories, as the percentage difference in performance between the two training sessions with a 22 ± 2 h-interval between them, i.e. between t3 (Er_*t3*_) on day 7, 13, or 20 and t1 (Er_*t1*_) on day 8, 14, or 21 (Vago et al. 2007). Next, to measure decision accuracy, we determined the difference in the number of entries into the blocked zones (gray zones, Fig. 3B) between the two reversal learning phases. Here, the encoding scores (En_*e*_), as illustrated below, was operationally defined in this study as the mean number of entries into a blocked path across the last two training sessions on a particular day (i.e. t2 and t3 on days 13 and 20), whereas the first training session 22 ± 2 h later was selected as retrieval score (E_*r*_) (Churchwell et al. 2010). The percentage difference (% Accuracy index) was then calculated. The following formulas were then used:

1. % Retrieval index = $$\left[ {\frac{{E{r_{t3}} - E{r_{t1}}}}{{E{r_{t3}}}}} \right] \times 100$$.

Er *= errors made*.

2. % Accuracy index = $$\left[ {\frac{{E{n_{t2}}+E{n_{t3}}}}{{E{n_e}+E{n_r}}}} \right] \times 100$$.

$$E{n_e}=\frac{{\left( {E{n_{t2}}+E{n_{t3}}} \right)}}{2}$$

En = *entry into the blocked path*.

En_*e*_ = *encoding scores for entry into the blocked path*.

En_*r*_ = *retrieval scores for entry into the blocked path*.

## Immunohistochemistry

Sixteen weeks after the AAV injection, Ncan and Control shRNA-injected mice were anesthetized using isoflurane and perfused transcardially with ice-cold phosphate-buffered saline (PBS) and fixed with 4% PFA for 10 min (Fig. 1A). Mice brains were then dissected, incubated in 4% PFA overnight, cryoprotected in 30% sucrose for 48 h, and frozen in 100% 2-methylbutane at −80 °C. The dissected brains were incubated in 4% PFA in PBS, cryoprotected in 30% sucrose in phosphate buffer (PB) for 48 h, and frozen in 100% 2-methylbutane at −80 °C. Slices of 50-µm thick coronal sections were kept floating in solution (1 part ethylene glycol, 1 part glycerin, 2 parts PBS, pH = 7.2) at 4 °C. For each staining, at least 2 sections per animal were used. The sections were first washed in 120 mM phosphate buffer (PB) at pH 7.2, then permeabilized with PB containing 0.5% Triton X-100 (Sigma, T9284) for 10 min at 37 °C. Then sections were blocked with a blocking solution (PB supplemented with 0.3% Triton X-100 and 5% normal goat serum, NGS, Gibco, 16210-064) for 1 h at 37 °C. Sections were then incubated with primary antibodies (for 20 h) and secondary antibodies (for 3 h) at 37 °C (Tables 1 and 2), and washed 3 times in PB after each incubation. Finally, sections were mounted on Superfrost slides with Fluoromount (Sigma, F4680), and confocal images were acquired with the Zeiss LSM 700 microscope.

**Table 1:** Full description of primary reagents

Reagent	Host Type	Dilution	Company	Reagent ID	Validation of specificity
Anti-PV	Mouse Monoclonal	1:500	Sigma-Aldrich	P3088	Specific binding to PV, not to other EF-hand proteins
Anti-PV	Chicken Polyclonal	1:500	Synaptic Systems	AB2619887	Peptide competition
Anti-vGAT	Guinea pig Polyclonal	1:500	Synaptic Systems	AB887873	Peptide competition
Anti-vGAT	Rabbit Polyclonal	1:500	Synaptic Systems	131,002	KO validated
Anti-PSD95	Mouse Monoclonal	1:250	Neuromab	73 − 028	KO validated
Anti-vGLUT1	Guinea pig Polyclonal	1:2000	Synaptic Systems	135,304	KO validated
Anti-Ncan	Sheep Polyclonal	1:100	R&D Systems	AF5800	KO validated
Anti-vGLUT2	Mouse Monoclonal	1:500	Abcam	AB79157	WB bands at expected size
Anti-CaMKII	Mouse Monoclonal	1:500	Abcam	AB22609	WB bands at expected size
WFA		1:1000	BioLogo/Vector Laboratories	B1355	GalNAc competition

The wild-type and Ncan KO mice were perfused as follows. Deep anesthesia was induced by isoflurane overdose, then animals were perfused transcardially, first with NaCl 0.9% for 1 min and then with PFA 4% in phosphate buffer (PB) 0.1 M for 20 min. After perfusion, brains were extracted and post-fixed by immersion in PFA 4% for 2 h. Then, brains were cryoprotected in 30% sucrose in PB 0.1 M and frozen. Afterward, brains were cut into 40-micron sections using a cryostat, and the sections were collected and stored at −20 °C. Sections were processed for fluorescence immunohistochemistry as follows. Slices were washed in saline phosphate buffer (PBS), then incubated for 2 h in 5% normal donkey serum (NDS, Gibco), 0.2% Triton-X100 in PBS to block nonspecific binding. After that, sections were incubated for 48 h at 4 °C with different primary antibodies (Table 1). Then, sections were washed and incubated with secondary antibodies for 2 h at room temperature (Table 2). Finally, sections were washed in 0.1 M PB, mounted, and cover-slipped with Vectashield fluorescence mounting medium (Vector Laboratories).

**Table 2:** Full description of secondary reagents

Reagent	Host	Label	Dilution	Company	Antibody ID
Anti-Chicken	Goat	Alexa 647	1:400	Life Technologies (Invitrogen)	A21449
Anti-Guinea pig	Goat	Alexa 555	1:400	Life Technologies (Invitrogen)	A11073
Anti-Mouse	Goat	Alexa 488	1:400	Life Technologies (Invitrogen)	A11029
Anti-Mouse	Goat	Alexa 405	1:1000	Life Technologies (Invitrogen)	A31553
Anti-Rabbit	Goat	Alexa 546	1:1000	Life Technologies (Invitrogen)	A11035
Anti-Mouse	Goat	Alexa 546	1:1000	Life Technologies (Invitrogen)	A11030
Anti-Guinea pig	Goat	Alexa 647	1:1000	Life Technologies (Invitrogen)	A21236
Anti-Sheep	Donkey	Alexa 546	1:1000	Life Technologies (Invitrogen)	A21098
Anti-Mouse	Goat	Alexa 546	1:1000	Life Technologies (Invitrogen)	A11030
Streptavidin		Alexa 647	1:1000	Life Technologies (Invitrogen)	S21374

### Data Acquisition, Processing, and Analysis

Two Control shRNA mice were excluded as outliers (ROUT method Q = 1%) in GraphPad Prism 8.0 (GraphPad Software Inc., La Jolla, USA) after the 2nd recency task, whereas one Ncan shRNA mouse died after the 1 st recency task. Additionally, one Control shRNA was further excluded from analysis due to very low GFP expression. For the vGLUT1 + puncta analysis, two Ncan shRNA sections were excluded since the sections and staining were of low quality. Overall, sections from 7 Control shRNA and 9 Ncan shRNA-treated mice were used for the IHC analysis. Regarding Ncan KO histology, all analyses were based on data from 5 wild-type mice and 6 Ncan KO mice, except for WFA fluorescence intensity, for which one Ncan KO mouse was excluded as an outlier (ROUT method, Q = 1%) in GraphPad Prism 8.0. Sections from Ncan shRNA-injectedmice were analyzed under a confocal Zeiss LSM 700 microscope, whereas Ncan KO mice were analyzed using a confocal Leica SPE microscope. Four z-stacks (up to 6 μm deep) were obtained at three different coronal levels (+ 2.2, + 1.9, and + 1.6 mm from Bregma, respectively). We used a 63x objective and 2x digital zoom for imaging perisomatic and neuropil puncta. The acquisition conditions for laser power, gain, and offset settings were maintained throughout all imaging sessions in temperature-controlled rooms (20 ± 2 °C) to compare fluorescence intensity between samples.

Quantification of synaptic puncta was performed using standardized image analysis procedures in Fiji (Schindelin et al. 2012). For each animal, multiple regions of interest from several infralimbic cortex sections were analyzed, and mean values were calculated per animal before statistical comparison between experimental groups. For the quantification of perisomatic puncta in shRNA-treated and Ncan KO mice, we manually outlined the profiles of 20–30 cell somata with clear cellular borders and quantified puncta using previously described macros (Guirado et al. 2018). Briefly, we defined a 1.5 μm band around somata as the region of interest (ROI). Then, the bands were duplicated, cleared outside, background subtracted with the rolling value of 50, and Gaussian blur with s-value of 1 and using the find maxima plugin in Fiji with a prominence of 5, size (0.3 to 1.0 µm^2^) and circularity (0.5–1.0), we measured the mean intensity along with the number of synaptic puncta. For neuropil analysis, we randomly selected 4 rectangular ROIs (100 µm^2^) per image and used the aforementioned procedure to measure fluorescent intensity and the number of synaptic puncta. All measurements were averaged per animal, and the resulting mean values were used for statistical analysis.

### Statistical Analysis

Statistical analysis was performed using GraphPad Prism 8.0 (GraphPad Software Inc., La Jolla, USA) and Statistica 8.0 (StatSoft, USA) software. The authors were blinded to the experimental group assignments and the specific protocol conditions throughout all experiments and subsequent statistical analyses to ensure unbiased data collection and interpretation. All data are shown as mean ± SD with n being the number of mice. The hypothesis that experimental distributions follow the Gaussian law was verified using Kolmogorov-Smirnov, Shapiro-Wilk, or D’Agostino tests. For pairwise comparisons, we used the Student’s t-test when the samples met the normality assumptions; otherwise, the Mann-Whitney test was employed. Spearman correlation coefficients were computed to estimate the association between the variables. In cases of multifactorial design of experiments, two- or three-way ANOVA was used. The p-values represent the level of significance as indicated in figures by asterisks (*p < 0.05, **p < 0.01, ***p < 0.001, and **** *p* < 0.0001) unless stated otherwise. The outcomes of the statistical tests in Ncan KO and Ncan shRNA-treated mice are provided in Supplementary Tables 1 and 2, respectively.

## Results

### Knockdown of Ncan Expression Impaired PNN Maintenance

To study the contribution of Ncan to the maintenance of PNNs around PV+ interneurons in vivo, we injected the already validated Ncan shRNA AAV (shRNA1 validated elsewhere (Baidoe-Ansah et al. 2025) into the infralimbic cortex (which is considered to be homologous to the dorsal part of the mPFC in humans) of young adult C57BL6J male mice. Using immunohistochemistry, we compared the perisomatic expression of Ncan and WFA labeling of PNNs around PV+ interneurons in the mPFC 16 weeks after AAV injection (Figs. 1B & C) and compared it to Ncan knockout mice (Figs. 1E & F). Analysis of mean fluorescent intensities of WFA and Ncan signals per animal confirmed that the expression of Ncan shRNA significantly reduced Ncan protein expression (*p* = 0.0050; unpaired t-test, Fig. 1C). This reduction was associated with a significant decrease in the WFA signal (*p* = 0.0167; Unpaired t-test, Fig. 1C). Moreover, we observed a significant positive relationship between the expression levels of Ncan and WFA in the GFP-positive cells infected with Ncan shRNA (Spearman coefficient of correlation rho = 0.559; *p* < 0.0001; Fig. 1D), but not with Control shRNA (Spearman coefficient of correlation rho = −0.033; *p* = 0.775; Fig. 1D), suggesting that the forced downregulation of Ncan impaired the maintenance of the molecular composition of PNNs. Then, we examined the effect of constitutive Ncan KO on WFA signal intensity in PNNs (Fig. 1E) and revealed a significant increase between groups (*p* = 0.0107; unpaired t-test, Fig. 1F), suggesting compensatory changes in PNN glycosylation in Ncan KO mice. In addition, we confirmed the specificity of the Ncan antibody, as previously described (Irala et al. 2024), by demonstrating a lack of Ncan expression in the PFC of Ncan KO mice (Fig. 1E).

### Impaired Temporal Order Recognition and Worsened Reversal Spatial Learning After Ncan Knockdown

Next, we aimed to determine the cognitive effects of Ncan knockdown in the PFC. Therefore, we measured two essential PFC-dependent cognitive functions, namely temporal order recognition memory and spatial reversal learning, by using the recency and the labyrinth (dry maze) tasks, respectively. Locomotor behavior and anxiety were controlled by subjecting mice to the open field test (Fig. 2A). Given the slow turnover of mature ECM components (Decaris et al. 2014; Matsubayashi et al. 2020), we performed tests 8 and 16 weeks after Ncan shRNA AAV delivery (Fig. 2A). In the open field test, the ratio of time spent in central to peripheral areas revealed no signs of anxiety/stress (shRNA x Time: *F* = 1.033; *p* = 0.3170; two-way ANOVA; Fig. 2D). Further, no effect of Ncan shRNA AAV on the distance traveled was observed 8 and 16 weeks after AAV injection (shRNA x Time: *F* = 0.00045; *p* = 0.3170; two-way ANOVA; Fig. 2E).

In the temporal order recognition (recency) task (Figure. 2 A), neither Ncan shRNA nor Control shRNA mice showed preferential exploration of the “old” object (first sampling phase) relative to the “recent” object (second sampling phase) at 8 weeks post-injection. By 16 weeks post-injection, however, Control shRNA mice spent significantly more time exploring the old object than the recent object (shRNA x Object x Time: *F* = 13.31; *p* = 0.0045; three-way ANOVA followed by Holm-Sidak’s post-hoc test, Fig. 2F). This pattern was further supported by discrimination ratio analysis, which confirmed a significant difference between Control shRNA and Ncan shRNA groups (shRNA x Time: *F* = 7.851; *p* = 0.0086; two-way ANOVA; Fig. 2G). Together, these findings suggest that Ncan loss in the PFC impairs temporal order recognition memory.

Then, we investigated the effects of Ncan knockdown on spatial learning and reversal spatial learning (relearning) in the Labyrinth, considering that the decision-making during relearning (cognitive flexibility) critically depends on the mPFC (Guise and Shapiro 2017) (Fig. 3A&B). Overall, there were no differences between groups in the distance traveled (*p* = 0.735; two-way RM ANOVA, Fig. 3C) and in the mean number of errors in making correct navigation decisions to reward location (*p* = 0.724; two-way RM ANOVA, Fig. 3D) during the learning and relearning phases. However, Ncan shRNA-treated mice traveled longer distances and made significantly more errors at the 2nd training session on day 13 during the 1 st relearning phase (*p* = 0.0406 and *p* = 0.0286, respectively; two-way ANOVA with Fisher’s LSD test, Fig. 3C&D). This observation suggests a potential decrease in short-term encoding and retrieval efficiencies due to Ncan shRNA, although this effect was lost in the 2nd relearning phase. We also estimated the retrieval index (as defined in the Methods section) between the last training session (t3) on the first day and the first session (t1) of the next day for each phase, by using the mean number of errors. During the learning phase, we observed no difference in the retrieval of spatial memory between t3 on day 7 and t1 on day 8 (47.50 ± 14.00% vs. 41.99 ± 21.69%; *p* = 0.8387; unpaired t-test, Fig. 3D) for both groups of animals. There was a weak tendency of impaired retrieval of long-term reversal spatial memory in Ncan shRNA, i.e., during the 1 st relearning phase (14.79 ± 8.55% vs. −3.83 ± 10.06%; *p* = 0.20881282; unpaired t-test, Fig. 3D). However, the retrieval performance of Ncan shRNA-treated mice showed a stronger tendency to be impaired during the 2nd relearning phase (32.05 ± 8.20% vs. −1.36 ± 14.09%; *p* = 0.0662; unpaired t-test, Fig. 3D). Additionally, we used the accuracy index (as defined in the Methods section) to measure the accuracy of the first decision-making step on where to go, and we found that mice injected with Ncan shRNA made more errors at the decision point by entering blocked paths in both relearning phases. This occurred 22 ± 2 h after mice relearned the new path to the new reward location, i.e. exactly at t1 on both day 14 (1st relearning-phase; 37.50 ± 18.30% vs. −7.41 ± 7.41%; *p* = 0.0294; Mann-Whitney, Fig. 3E) and day 21 (2nd relearning-phase; 46.40 ± 11.20% vs. 13.60 ± 12.32%; *p* = 0.0699; unpaired t-test, Fig. 3E). This result suggests that Ncan may be essential for the accurate retrieval of reversal spatial memory after its overnight consolidation.

### Reduced GABAergic Innervation After Ncan Knockdown

**Fig. 4:**
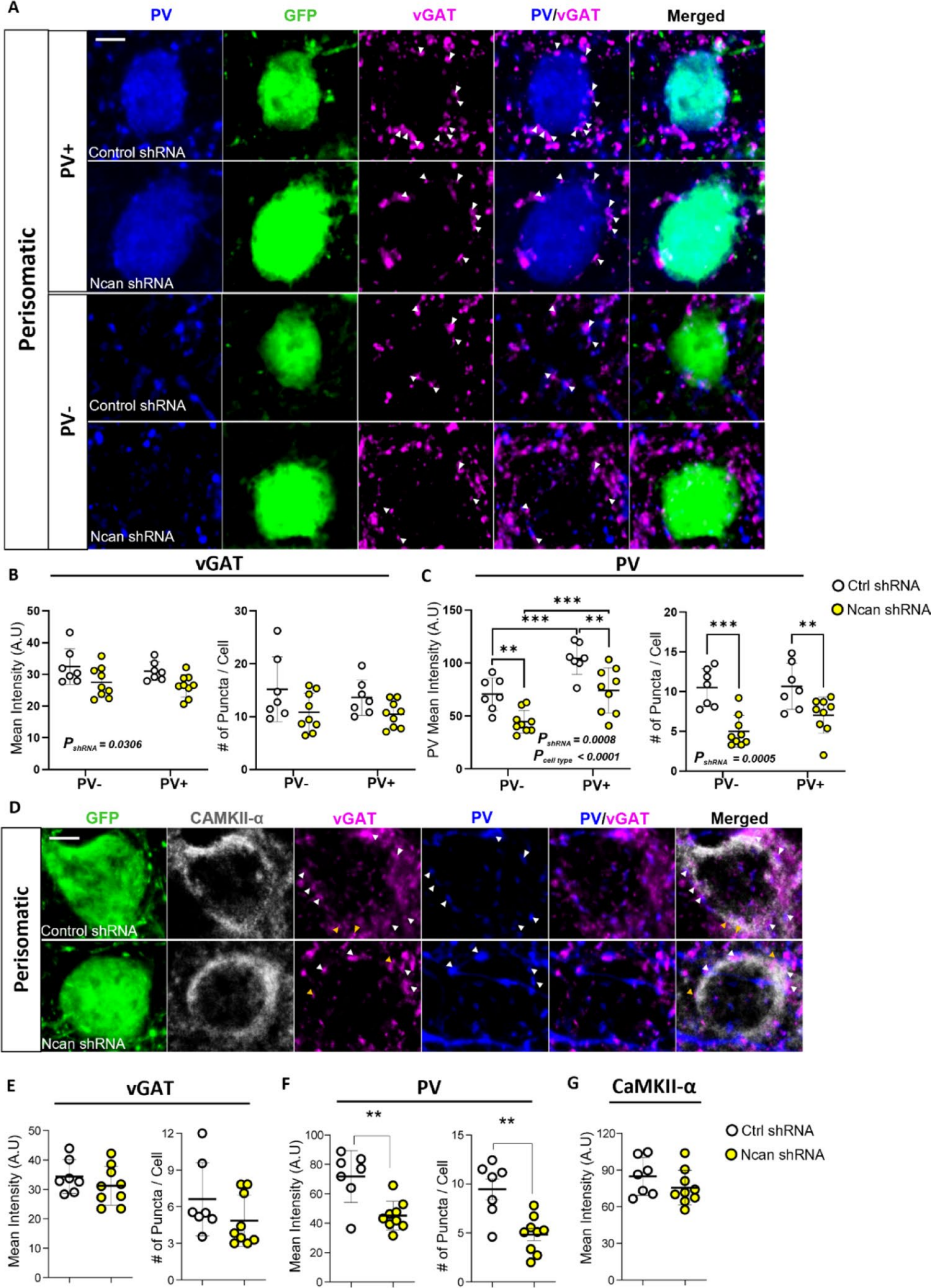
Ncan knockdown reduces perisomatic GABAergic innervation of PV+ interneurons. **A** Representative images showing the expression of vGAT+ puncta in shRNA-infected PV- and PV+ neurons in the PFC. White arrowheads point to vGAT+ puncta. Scale bar, 5 μm. **B**,** C** In Ncan shRNA-treated mice, the perisomatic fluorescence intensity of vGAT + and PV+ puncta was significantly reduced relative to Control shRNA. There was also a significant reduction in the density of perisomatic PV+ puncta on PV + and PV- neurons. Control shRNA (2100 PV+ puncta and 1758 vGAT+ puncta from 110 PV- neurons; 992 PV+ puncta and 735 vGAT+ puncta from 49 PV+ neurons from 7 mice), Ncan shRNA (1472 PV+ puncta and 1020 vGAT+ puncta from 148 PV- neurons; 1074 PV+ puncta and 850 vGAT+ puncta from 81 PV+ neurons from 9 mice), two-way ANOVA with Holm-Sidak’s post-hoc test. **D** Representative images showing the expression of vGAT + and PV+ puncta on shRNA-infected CAMKII-α + neurons. Scale bar, 5 μm. **E** Changes in vGAT mean intensity and puncta in KO mice. **F** PV mean intensity decreased after Ncan downregulation. **G** No changes in CAMKII-α mean intensity between groups. Control shRNA (600 PV+ puncta and 427 vGAT+ puncta from 62 neurons from 7 mice), Ncan shRNA (432 PV+ puncta and 402 vGAT+ puncta from 87 neurons from 9 mice), unpaired t-test with Welch’s correction. Bar graphs show mean ± SD values. *p < 0.05, **p < 0.01, ***p < 0.001 and **** *p* < 0.0001 represent significant differences

Prompted by the effects of Ncan dysregulation on temporal recognition memory and reversal spatial learning, as observed in this study, we then analyzed the possible cellular and molecular underpinnings. We used immunohistochemistry to quantify synaptic changes around PFC inhibitory and excitatory neurons in Ncan shRNA- and Control shRNA-treated mice, as previous studies have suggested a potential role of Ncan in perisomatic innervation (Sullivan et al. 2018; Schmidt et al. 2020). Using antibodies against the vesicular GABA transporter (vGAT) (Fig. 4A) to study perisomatic GABAergic innervation of PV + and PV- neurons, we observed a significant reduction in vGAT fluorescence intensity in Ncan shRNA-treated mice (shRNA: *F* = 5.783; *p* = 0.0306; two-way ANOVA followed by Holm-Sidak’s post-hoc test, Fig. 4B), whereas the density of perisomatic vGAT+ puncta on both PV + and PV- neurons was unchanged (shRNA: *F* = 4.164; *p* = 0.0756; two-way ANOVA followed by Holm-Sidak’s post-hoc test, Fig. 4B). In the same animals, PV+ puncta, representing presynaptic boutons and axonal varicosities arising from PV+ interneurons, showed reduced fluorescence intensity across PV- and PV+ neurons (shRNA: *F* = 18.36; *p* = 0.0008; cell type: *F* = 42.73; *p* < 0.0001; two-way ANOVA followed by Holm-Sidak’s post-hoc test, Fig. 4C) accompanied by a decrease in puncta density (shRNA: *F* = 20.11; *p* = 0.0005; two-way ANOVA followed by Holm-Sidak’s post-hoc test, Fig. 4C).

Next, we quantified the perisomatic vGAT+ puncta on CAMKII+ pyramidal neurons (Fig. 4D), which constitute the majority of PV- cells, and observed no difference in the intensity and the density of vGAT+ puncta between shRNA groups (*p* = 0.3354 and 0.1359, respectively; unpaired t-test and Mann-Whitney, respectively, Fig. 4E). On the other hand, knockdown of Ncan decreased the fluorescent intensity and the density of perisomatic PV+ puncta on CAMKII+ pyramidal neurons (*p* = 0.0018 and 0.0014, respectively; unpaired t-test, Fig. 4F). We did not detect any difference in the fluorescent intensity of CaMKII in the GFP+ somata between both shRNA groups (*p* = 0.2330; unpaired t-test, Fig. 4G).

### Increased Glutamatergic Innervation After Ncan Knockdown

**Fig. 5:**
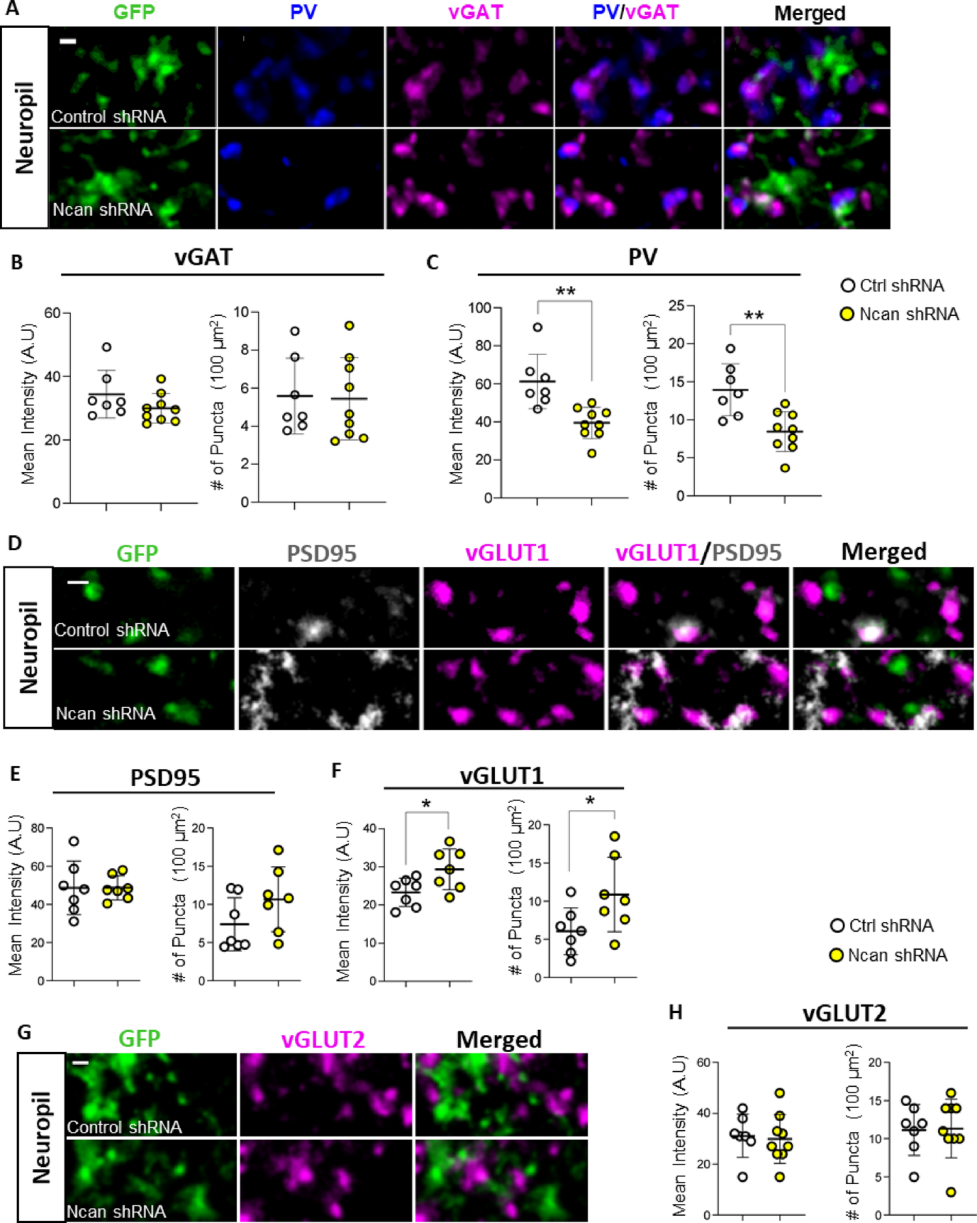
Ncan knockdown decreases GABAergic innervation and increases glutamatergic innervation by vGLUT1 + terminals in the neuropil. **A** Representative confocal images showing vGAT + and PV+ puncta in the neuropil of the prefrontal cortex in Control shRNA and Ncan shRNA-treated mice. Scale bar, 1 μm. Quantification was performed using randomly selected rectangular regions of interest (ROIs; 100 μm²) per image. **B** Quantification of the mean fluorescence intensity and density of vGAT+ puncta revealed no significant differences between groups. **C** In contrast, the fluorescence intensity and density of PV+ puncta were reduced in Ncan shRNA-treated mice compared with Control shRNA-treated mice. Control shRNA (3974 PV+ puncta and 1571 vGAT+ puncta from 281 ROIs from 7 mice), Ncan shRNA (3437 PV+ puncta and 2058 vGAT+ puncta from 394 ROIs from 9 mice), unpaired t-test with Welch’s correction (**p < 0.01). **D** Representative images showing vGLUT1 + and PSD95 + puncta in the PFC neuropil. Scale bar, 1 μm. **E** Quantification revealed no significant differences in the fluorescence intensity or density of PSD95 + puncta between groups. **F** The fluorescence intensity and density of vGLUT1 + puncta were increased in Ncan shRNA-treated mice relative to Control shRNA-treated mice. Control shRNA (507 vGLUT1 + puncta and 850 PSD95 + puncta from 152 ROIs from 7 mice), Ncan shRNA (828 vGLUT1 + puncta and 1221 PSD95 + puncta from 153 ROIs from 7 mice), unpaired t-test with Welch’s correction (**p < 0.01). **(G)** Representative images of vGLUT2 + puncta in the PFC neuropil. **(H)** Quantification of vGLUT2 + puncta revealed no significant differences between groups. Control shRNA (2316 vGLUT2 + puncta from 317 ROIs from 7 mice), Ncan shRNA (2338 vGLUT2 + puncta from 326 ROIs from 9 mice), unpaired t-test with Welch’s correction. Bar graphs show mean ± SD values

In the neuropil, Ncan depletion did not affect the intensity or number of vGAT+ puncta (*p* = 0.1710 and *p* = 0.8940, respectively; unpaired t-test; Fig. 5B). By contrast, we found reduced fluorescent intensity and density of PV+ puncta (*p* = 0.0017 and *p* = 0.0027, respectively; unpaired t-test, Fig. 5C). Given the presence of Ncan in the perisynaptic ECM of excitatory synapses, we aimed to examine the effects of Ncan depletion on this synapse type in the PFC neuropil. Hence, we analyzed the expression of postsynaptic density protein 95 (PSD95), a critical scaffolding protein for anchoring excitatory synaptic proteins, including NMDA receptors (Lim et al. 2003; Won et al. 2016). No difference was observed in the neuropil fluorescent intensity along with the number of PSD95 + puncta between shRNA groups (*p* = 0.9918 and *p* = 0.1649, respectively; unpaired t-test and Mann-Whitney, respectively, Fig. 5E). Then, we analyzed the expression of presynaptic vesicular glutamate transporters (vGLUT) in the neuropil. The vGLUT family includes vGLUT1, vGLUT2, and vGLUT3, which are essential for the uptake of L-glutamate into synaptic vesicles of excitatory neurons. VGLUT1 is expressed in synapses of cortical origin, while VGLUT2 is exclusively expressed in extracortical inputs (Cheng et al. 2011; Nordenankar et al. 2015). The fluorescent intensity and the density of vGLUT1 + puncta in the neuropil area were significantly higher in Ncan shRNA- compared to Control shRNA-treated mice (*p* = 0.0300 and *p* = 0.0474, respectively; unpaired t-test, Fig. 5F). In contrast, the fluorescent intensity and the density of vGLUT2 + puncta (Fig. 5G) were not different between shRNA groups (*p* = 0.7889 and *p* = 0.9186, respectively; unpaired t-test, Fig. 5H).

### Effect of Ncan Knockout on Inhibitory and Excitatory Innervation

**Fig. 6:**
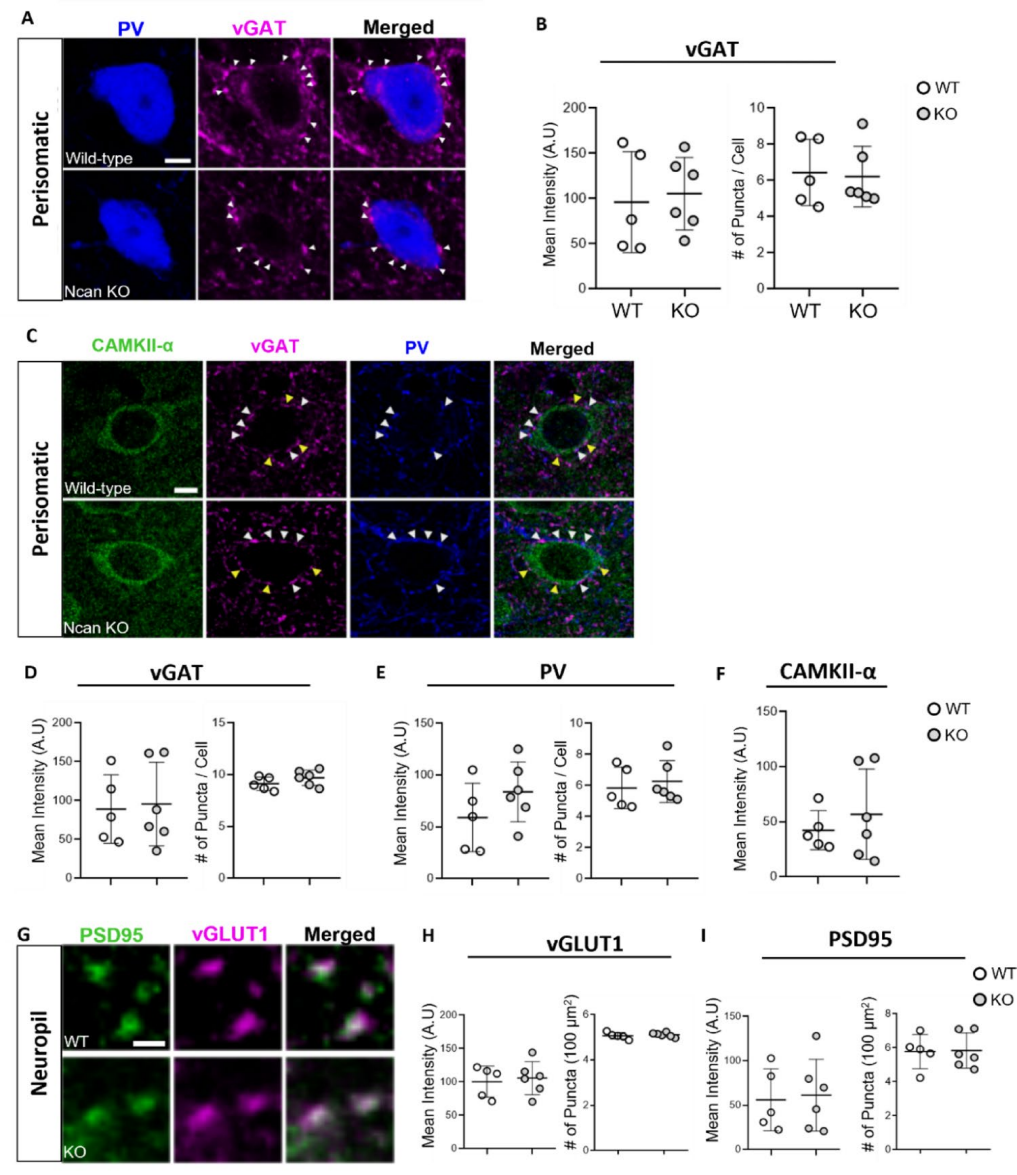
No effects of Ncan knockout on GABAergic innervation of excitatory pyramidal neurons. **A** Representative images showing vGAT+ puncta contacting the somata of PV+ interneurons in 3-month-old Ncan KO mice or age-matched wild-type mice. White arrowheads point to vGAT+ puncta. Scale bar, 5 μm. **B** Quantification of the mean immunofluorescence intensity and the number of vGAT+ puncta (799 VGAT+ puncta on 118 PV+ cells from 5 mice), and Ncan KO mice (769 VGAT+ puncta on 130 PV+ cells from 6 mice), unpaired t-test for immunofluorescence intensity and Mann-Whitney test for vGAT puncta. **C** Representative images showing vGAT+ puncta in CAMKII-α positive cells, Scale bar, 5 μm. **D–F** Quantification of the mean immunofluorescence intensity and the number of vGAT+ **(D)**, PV **(E)**, and CAMKII-α **(F)** in WT (900 PV + and 1410 VGAT+ puncta on 156 CAMKII-α + cells from 5 mice), and KO mice (1163 PV + and 1809 VGAT+ puncta on 185 CAMKII-α + cells from 6 mice), unpaired t-test. **G** Representative images of vGLUT1 + and PSD95 + in the neuropil area of WT and Ko mice. **H**,** I** Quantification of the mean immunofluorescence intensity and the number of vGLUT1+ **(H)** and PSD95+ **(I)** in WT (5262 PSD95 + and 4618 vGLUT1 + punctafrom 10 ROIs of 5 mice), and KO mice (6381 PSD95 + and 5590 vGLUT1 + puncta from 10 ROIs in 6 mice). Unpaired t-test

To test whether this phenotype is also observed after constitutive Ncan depletion, we examined perisomatic GABAergic innervation in Ncan KO mice (Fig. 6A). Conversely to Ncan shRNA mice, we did not find significant alterations in the perisomatic density of vGAT puncta, nor their fluorescence intensity on PV+ cells of Ncan KO mice as compared to wild-type mice (*p* = 0.8298 and *p* = 0.7528, respectively; unpaired t-test, Fig. 6B). To analyze the effects of the genetic depletion of Ncan on the perisomatic inhibition of pyramidal neurons, we quantified the density of perisomatic puncta positive for vGAT and PV on CaMKII+ pyramidal neurons (Fig. 6C). Neither the density of vGAT+ puncta nor their fluorescence intensity was significantly affected in Ncan KO mice (*p* = 0.2058; unpaired t-test, *p* = 0.8329; unpaired t-test, respectively; Fig. 6D). No significant difference between wild-type and Ncan KO mice was found regarding the density of PV+ puncta and their fluorescence intensity (*p* = 0.6244; unpaired t-test, *p* = 0.2154; unpaired t-test, respectively; Fig. 6E). Finally, we analyzed CAMKII fluorescence intensity in pyramidal cell somata and found no significant differences between groups (*p* = 0.4816; unpaired t-test; Fig. 6F). Additionally, we analyzed the effect of constitutive Ncan knockout on vGLUT1 + and PSD95 + puncta in the neuropil (Fig. 6G). No significant differences between Ncan KO and wild-type mice were detected in the fluorescence intensity and the density of vGLUT1 + puncta (*p* = 0.727 and *p* = 0.559, respectively; unpaired t-test with Welch’s correction, Fig. 6H) and PSD95 + puncta (*p* = 0.829 and *p* = 0.9238, respectively; unpaired t-test, Fig. 6I).

## Discussion

Using shRNA-mediated knockdown of Ncan in the mPFC, we observed alterations in PNN-associated markers together with mild but consistent impairments in temporal order recognition memory and aspects of reversal spatial learning in the labyrinth task. Moreover, we observed a reduction in expression of immunohistochemical markers associated with GABAegric innervation by PV+ interneurons and an increase in the density of vGLUT1 + presynapses in the PFC neuropil.

### Ncan Knockdown and PNN Alteration

PNNs form and mature postnatally, from around P14 to P50, in mice, and Ncan expression follows a similar pattern (Pizzorusso et al. 2002). The composition of lecticans within PNNs has been shown to vary between brain regions (Lensjo et al. 2017; Fawcett et al. 2019). Acan, HAPLN1, TnR, and phosphacan, but not Bcan, have recently been identified as essential contributors to PNN formation (Morawski et al. 2014; Suttkus et al. 2014; Eill et al. 2020). Additionally, a recent analysis of Ncan KO mice implicated Ncan as a major contributor to PNN integrity in the MNTB, where the authors reported alterations in expression of other ECM molecules, like decreased mRNA levels of HAPLN1 and Bcan (Schmidt et al. 2020). Moreover, we recently reported in an in vitro study that Ncan reduction remodels PNNs (Baidoe-Ansah et al. 2025). In the present work, we also show that Ncan contributes to PNN maintenance in vivo. Treatment with shRNA against Ncan resulted in about 25% reduction of Ncan immunoreactivity in PNNs. This reduction is in line with the decrease in Ncan mRNA observed in our previous in vitro study (Baidoe-Ansah et al. 2025) and was accompanied by less prominent WFA-staining of PNNs, which binds to CSPG glycosaminoglycan chains (Dityatev et al. 2007). This reduction in WFA labeling likely reflects changes in the molecular composition or glycosylation state of PNN components, rather than a net decrease in PNNs. Possible reasons for the rather small reduction in Ncan immunoreactivity include the expression of Ncan in non-infected neurons within the mPFC or in cells from different brain regions projecting to the mPFC (Hoover and Vertes 2007) or the production and secretion of Ncan by astrocytes (Galtrey et al. 2008; Fawcett et al. 2019). Notably, the AAV2/DJ viral particles used in our study predominantly target neurons in vivo (Jollé et al., 2019); hence, it is likely that the major fraction of the depleted Ncan in the medial prefrontal cortex of Ncan shRNA-treated group was of neuronal origin. Remarkably, we observed a linear relationship between reduced Ncan and WFA signals after Ncan knockdown. From our previous report, the striking effects of Ncan knockdown on mRNA expression levels of other PNN molecules, such as downregulation of HAPLN1 and Bcan, along with upregulation of Acan (Baidoe-Ansah et al. 2025), also reported in Ncan KO mice (Schmidt et al. 2020), support the on-target efficacy of our knockdown vectors (Taxman et al. 2006; Okuda et al. 2014). Contrary to the KO study in the MNTB (Schmidt et al. 2020), the PNN structure was not strongly disrupted in the mPFC of Ncan-knockdown mice compared to wild-type, which may be either due to region-specific differences in PNN composition (Morikawa et al. 2017) or the level of Ncan reduction (100% vs. 25%). In summary, these data indicate that neuronal Ncan is an essential component of PNNs around PV+ interneurons in the mPFC. The differences between adult, region-specific Ncan knockdown and constitutive Ncan knockout mice are consistent with the idea that genetic deletion can engage compensatory mechanisms during development, whereas acute depletion in later developmental stages may reveal functions that are otherwise masked. Supporting this interpretation are data showing very early expression of Ncan gene in development, starting around E12.5 (Zhou et al. 2001). Also, in primary cortical neuronal cultures, we recently found that the expression of ECM constituents such as brevican and the link proteins *Hapln1* and *Hapln4* exhibited differential regulation depending on whether Ncan was acutely reduced by shRNA or constitutively deleted. These findings demonstrate that Ncan perturbation can drive homeostatic adjustments within the ECM/PNN molecular meshwork and provide a plausible framework for interpreting why synaptic alterations observed after adult knockdown are not necessarily recapitulated in the constitutive knockout model. Together, the knockdown–knockout comparison therefore highlights compensation as a key outcome of the present study.

### Ncan Depletion Results in Mild Impairments in mPFC-Dependent Cognitive Functions

Neural ECM remodeling has been shown to impact cognition, both positively and negatively. For example, in TnR KO mice, ECM attenuation enhanced reversal learning in the Morris water maze and olfactory discrimination task (Morellini et al. 2010), as well as working memory (Sykova et al. 2005). Also, digestion of ECM with hyaluronidase improved reversal learning (Happel et al. 2014) and glycosidase treatment with chABC in the perirhinal cortex enhanced object recognition memory (Romberg et al. 2013). On the flip side, reduced LTP in the hippocampal CA1 occurred in both TnR KO (Bukalo et al. 2001) and TnC KO mice (Evers et al. 2002), correlating with impaired fear extinction in the latter. Also, Bcan KO shows impaired hippocampal-dependent LTP without obvious cognitive deficits (Brakebusch et al. 2002). However, intra-hippocampal injection of hyaluronidase impaired LTP and contextual fear memory (Kochlamazashvili et al. 2010; Senkov et al. 2014). Additionally, the digestion of ECM in the visual cortex disrupted recall of visual fear memory (Thompson and Chen 2017) and, more importantly, in the mPFC, although in rats. Moreover, injection of chABC into the mPFC of rats resulted in below-chance performance in the cross-modal object recognition task (Paylor et al. 2018) and an impairment of LTP (Shi et al. 2019). In the mPFC of anesthetized mice, chABC injection decreased gamma activity, a neuronal oscillation dependent on PV+ cells, which is essential for various cognitive tasks involving this neocortical region (Carceller et al. 2020).

In the present study, ECM attenuation, induced by reduced Ncan expression, impaired temporal order recognition memory. Moreover, we found Ncan knockdown to negatively impact the consolidation of long-term reversal spatial memory (formed 22 ± 2 h after the initial reversal learning phase) as well as the decision accuracy estimated by the retrieval index. This method relies on the mean number of errors, as studies have shown this measure to be the more effective metric compared to latency to reward (Maei et al. 2009) to measure cognition in labyrinth tasks (Rogers and Kesner 2003; Lee and Kesner 2004; Vago et al. 2007; Churchwell et al. 2010). Reduced Ncan expression in the mPFC did not influence spatial memory acquisition and recall during the learning phase on the same day, but only during the memory tests on the next day. This result is thus in line with a previous study documenting reduced remote memory in the 24-hour delayed retrieval of verbal memories in healthy human Ncan rs1064395 risk allele carriers, and with the finding that these individuals showed reduced PFC gray matter density (Assmann et al., 2021). The subtle effects on reversal of spatial memory observed after Ncan knockdown in our study are likely to reflect a small reduction in Ncan expression. Another study in humans highlights similar functions of the mPFC, where different retrieval patterns were observed overnight relative to same-day memories (Ezzyat et al. 2018). These findings suggest that the integrity of the neural ECM in the mPFC may be essential for consolidating updated spatial memories. One possible interpretation is that alterations in PNN composition following Ncan depletion may influence mechanisms involved in memory consolidation (Gogolla et al. 2009; Leon et al. 2010). Also, the mPFC has been demonstrated to be critical for the retrieval of remote memories, as well as for the consolidation and recall of recent memories (Leon et al. 2010), and these neural processes are PNN-dependent.

### Ncan Reduction and Changes in Synaptic Innervation

Changes in synaptic strength have been shown to contribute to learning and memory through various molecular mechanisms such as those regulating long-term depression (LTD) and LTP. LTP is produced when N-methyl-D-aspartate (NMDA) receptors and Ca^2+^ voltage-dependent channels are activated as a result of presynaptic release of neurotransmitters (e.g., glutamate) and postsynaptic depolarization (Mayford et al. 2012; Langille and Brown 2018). This process depends on the balanced interaction of excitation and inhibition (E/I balance) (Tao et al. 2014; Kirkwood 2015), whereby variation in this balance affects basic cortical functions and may impair mPFC-dependent learning (Yizhar et al. 2011). Moreover, the E/I balance is found to be critical in several psychiatric disorders such as autism and schizophrenia (Kirkwood 2015). Interestingly, ECM modulation has been shown to affect the synaptic E/I balance in various model systems. For example, studies in TnR KO mice showed that deficiency in this ECM glycoprotein impairs formation of perisomatic GABAergic synapses on pyramidal neurons, and as a result, decreased perisomatic inhibition in the CA1 region (Nikonenko et al. 2003; Saghatelyan et al. 2004) and enhanced the basal excitatory transmission, resulting in a shift in the E/I balance (Saghatelyan et al. 2004). Also, a strong effect of the ECM on the balance between inhibitory and excitatory synapses has been observed in quadruple KO mice, where the loss of TnR, TnC, Bcan, and Ncan increased the number of excitatory synapses and reduced inhibitory synapses (Gottschling et al. 2019). Interestingly, the expression of glutamic acid decarboxylase (GAD), the enzyme that synthesizes the inhibitory neurotransmitter gamma-aminobutyric acid, depends on the ECM (Schmidt et al. 2020). As such, analysis of Ncan KO mice revealed reduced expression of GAD65/67, consistent with findings that enzymatic degradation of ECM decreased the density of cortical GABAergic perisomatic synapses (Sullivan et al. 2018; Schmidt et al. 2020).

Other studies have found that PNN depletion decreased the density of puncta immunopositive for inhibitory markers in the cortical neuropil, or specifically in the perisomatic region of PV+ cells (Carceller et al. 2020; Irala et al. 2024; Lensjo et al. 2017; Sullivan et al. 2018). In line with these studies, we observed that Ncan knockdown significantly decreased the intensity of vGAT puncta and the number of PV+ presynaptic puncta associated with PV+ interneurons. This observation might explain the findings from our previous in vitro study using the same shRNA, in which we revealed increased expression of activity-dependent c-Fos and FosB genes and elevated spontaneous synaptic activity (Baidoe-Ansah et al. 2025). The underlying molecular mechanism dissected in in vitro studies may involve inhibition of NCAM-EphA3 association by Ncan’s interaction with the immunoglobulin-like domain 2 (Ig2) of NCAM (Sullivan et al. 2018).

However, knockdown of Ncan also upregulated vGLUT1 (although not vGLUT2) immunoreactive puncta. In contrast to this finding, a recent study reports no change in excitatory synapse formation in vitro, neither in Ncan knockout cultures nor after Ncan addition in glia-free cultures (Irala et al. 2024). However, another study revealed that Ncan inhibited Semaphorin 3 (SEMA3)-dependent spine pruning (which happens mostly at excitatory postsynaptic sites), and consequently, Ncan depletion could have the opposite effect, thereby increasing the number of spines and excitatory synapses (Mohan et al. 2018). Additionally, previous studies have shown that vGLUT2 expression is independent of vGLUT1 upregulation (Wojcik et al. 2004), consistent with a specific effect of Ncan knockdown on vGLUT1 + presynapses. Overall, these data suggest that Ncan knockdown is associated with alterations in synaptic connectivity within the PV+ cell network and modulation of excitatory input to principal cortical cells, which may change the E/I balance in the mPFC in favor of excitation.

A limitation of the present study is that the synaptic alterations were assessed using well-established immunohistochemical markers of inhibitory and excitatory synapses, which provide structural correlates of synaptic connectivity but do not directly measure synaptic function. Therefore, the observed reductions in perisomatic GABAergic markers and increases in vGLUT1-positive puncta should be interpreted as indicative of altered synaptic organization rather than direct evidence for functional changes in excitatory–inhibitory balance. Our previous work revealed elevated synaptic activity following knockout of Ncan in dissociated cultures (Baidoe-Ansah et al. 2025). Future studies combining Ncan manipulation in vivo with electrophysiological recordings or two-photon calcium imaging will be important to determine how these structural changes influence neuronal and network activity in the mPFC. The second limitation of our work is that while the present findings reveal parallel alterations in synaptic organization and PFC-dependent behavior after Ncan knockdown, the data do not establish a direct causal relationship between these phenomena. Instead, the observed synaptic changes should be considered as candidate structural mechanisms that may contribute to the behavioral phenotype.

## Conclusion

Overall, our findings support the idea that ECM composition in the mPFC contributes to synaptic organization and may influence cognitive processes such as temporal memory and cognitive flexibility.

## Supplementary Information

Below is the link to the electronic supplementary material.

Supplementary Material 1

Supplementary Material 2

## Data Availability

Statistical analysis data is provided within the manuscript. Raw or analyzed data during the current study will be available from the corresponding authors on reasonable request.
